# Affinities of human NMDA receptor autoantibodies: implications for disease mechanisms and clinical diagnostics

**DOI:** 10.1007/s00415-018-9042-1

**Published:** 2018-09-05

**Authors:** Lam-Thanh Ly, Jakob Kreye, Betty Jurek, Jonas Leubner, Franziska Scheibe, Johannes Lemcke, Nina Kerstin Wenke, Sebastian Momsen Reincke, Harald Prüss

**Affiliations:** 1 German Center for Neurodegenerative Diseases (DZNE) Berlin, Berlin, Germany; 2 Department of Neurology and Experimental Neurology, Charité-Universitätsmedizin Berlin, CharitéCrossOver (CCO), R. 4-334, Charitéplatz 1, 10117 Berlin, Germany; 3 Department of Neurosurgery, Unfallkrankenhaus Berlin, Berlin, Germany

**Keywords:** NMDA receptor encephalitis, Antibody affinity, Human monoclonal antibody, Cerebrospinal fluid, Flow cytometry

## Abstract

Anti-*N*-methyl-d-aspartate receptor (NMDAR) encephalitis is a common autoimmune encephalitis presenting with psychosis, dyskinesias, autonomic dysfunction and seizures. The underlying autoantibodies against the NR1 subunit are directly pathogenic by disrupting synaptic NMDAR currents. However, antibody titers correlate only partially with the clinical outcome, suggesting the relevance of other factors such as antibody affinity. We thus determined the binding curves of human monoclonal autoantibodies and patients’ cerebrospinal fluid (CSF) against NR1-expressing HEK293 cells using flow cytometry. Antibody affinity was highly variable with binding constants (half-maximal concentration, *c*_50_) ranging from 1 to 74 µg/ml for monoclonal antibodies. Comparing values of individual monoclonal antibodies with human CSF samples suggested that the CSF signal is predominantly represented by higher-affinity antibodies, potentially in a concentration range of NR1 antibodies between 0.1 and 5 µg/ml, roughly reflecting 1–10% of total CSF IgG in NMDAR encephalitis. Binding curves further depended on the CSF composition which must be considered when interpreting established clinical routine assays. Normalization of measurements using reference samples allowed high reproducibility. Accurate and reproducible measurement of NR1 antibody binding suggested that biophysical properties of the antibody might contribute to disease severity. Normalization of the data can be an elegant way to allow comparable inter-laboratory quantification of CSF NR1 antibody titers in autoimmune encephalitis patients, a prerequisite for use as surrogate markers in clinical trials. Based on our calculations, low-affinity antibodies can easily remain undetected in routine cell-based assays, indicating that their relation to clinical symptoms should be analyzed in future studies.

## Introduction

Anti-*N*-methyl-d-aspartate receptor (NMDAR) encephalitis is a common autoimmune encephalitis presenting with psychosis, amnesia and dyskinesias, often progressing to severe autonomic dysfunction, seizures and reduced levels of consciousness requiring prolonged intensive care treatment [7]. The underlying autoantibodies target the aminoterminal domain of the NR1 subunit of NMDAR, and intrathecal injection of purified IgG from patients with NMDAR encephalitis led to behavioral abnormalities in mice compatible with the human disease [5]. Finally, the cloning, recombinant production and functional testing of cerebrospinal fluid (CSF)-derived monoclonal antibodies against NR1 confirmed that the antibodies are directly pathogenic by down-regulating neuronal NMDAR and disrupting synaptic NMDAR currents [2, 4].

Despite some intra-individual correlation between CSF NR1 antibody titers and clinical course in patients with good outcome [1], the titer correlation between different patients is poor, i.e., patients requiring several months of ICU therapy including mechanical ventilation may have lower NR1 antibody titers than patients with mild disease. This finding suggests the relevance of other parameters which could include biophysical properties of the antibody itself, such as antibody affinity or epitope binding, or antibody-independent factors, such as the patient-specific glutamate receptor turnover.

We therefore aimed to determine the affinity of human autoantibodies against NR1, which has become technically possible only after the generation of a panel of patient-derived monoclonal NR1 antibodies [2]. Antibody affinity defines the strength of interaction between an epitope (in this case the NMDAR) and the antigen-binding site of an antibody. The higher the affinity, the more the antibody is bound to its antigen at equilibrium. Thus, a high-affinity NR1-reactive autoantibody could potentially be much more pathogenic compared to a low-affinity autoantibody. For this, we applied binding analyses of human monoclonal antibodies and patients’ CSF against NR1-expressing HEK293 cells with flow cytometry, asking for the binding curves of individual antibodies and whole CSF, and estimation of specific NR1 antibody concentrations in human samples.

## Methods

### Monoclonal antibodies

Five monoclonal human NR1 antibodies from three patients (#003-102, #007-124, #007-168, #007-169, #008-218) were selected from a panel of CSF-derived monoclonal antibodies of patients with typical NMDAR encephalitis described previously [2]. Antibodies were generated by single antibody-secreting cell cloning of full-length immunoglobulin G (IgG) variable heavy and light chain genes and showed the characteristic binding to NR1-expressing human embryonic kidney (HEK293) cells and to murine brain sections. Isotype-matched non-NR1 control antibodies were derived from the same study, including an antibody not reactive to human tissues (#mGo53) [8] and an astrocyte-reactive human antibody (#011-123) [2].

### Flow cytometry (FACS)-based assay using monoclonal antibodies

IgG concentrations of the monoclonal human antibodies were determined with ELISA (Mabtech). Serial dilutions of each antibody were prepared in a 96-well plate, containing 80 µl per well, giving a final concentration of 300–0.001 µg/ml in FACS buffer (1% heat-inactivated fetal bovine serum (FCS) in phosphate-buffered saline (PBS)).

HEK293 cells were transfected with an NR1–EYFP plasmid which encodes the NMDAR NR1 subunit and enhanced yellow fluorescent protein (EYFP) without stop codon plus a geneticin resistance gene. The NR1–EYFP fusion protein was expressed in culture in DMEM supplemented with 10% FCS, 100 U/ml penicillin, 100 U/ml streptomycin and 800 µg/ml geneticin. For control experiments, EYFP was transiently transfected without NR1. HEK293 cells were harvested after washing with PBS and detachment with 0.5% trypsin/EDTA, washed again with PBS, centrifuged at 1700 RPM for 5 min and resuspended in ice-cold FACS buffer at a concentration of 15,000 cells/µl. 20 µl was added to the 96-well plate for a total of 300,000 cells/well and mixed with the monoclonal antibody solution.

Cells were incubated on ice for 45 min, washed with FACS buffer and centrifuged. After removing the supernatant, the secondary antibody (goat anti-human Alexa Fluor-647 [Life Technologies], 1:400 in FACS buffer) was added and incubated on ice for 20 min. Washing steps were repeated, cells resuspended in FACS buffer and transferred into FACS tubes placed on ice until FACS measurement on a BD FACS-Canto-II machine. The entire experiment was independently replicated 5 times.

### Affinity calculation

**Fig. 1:**
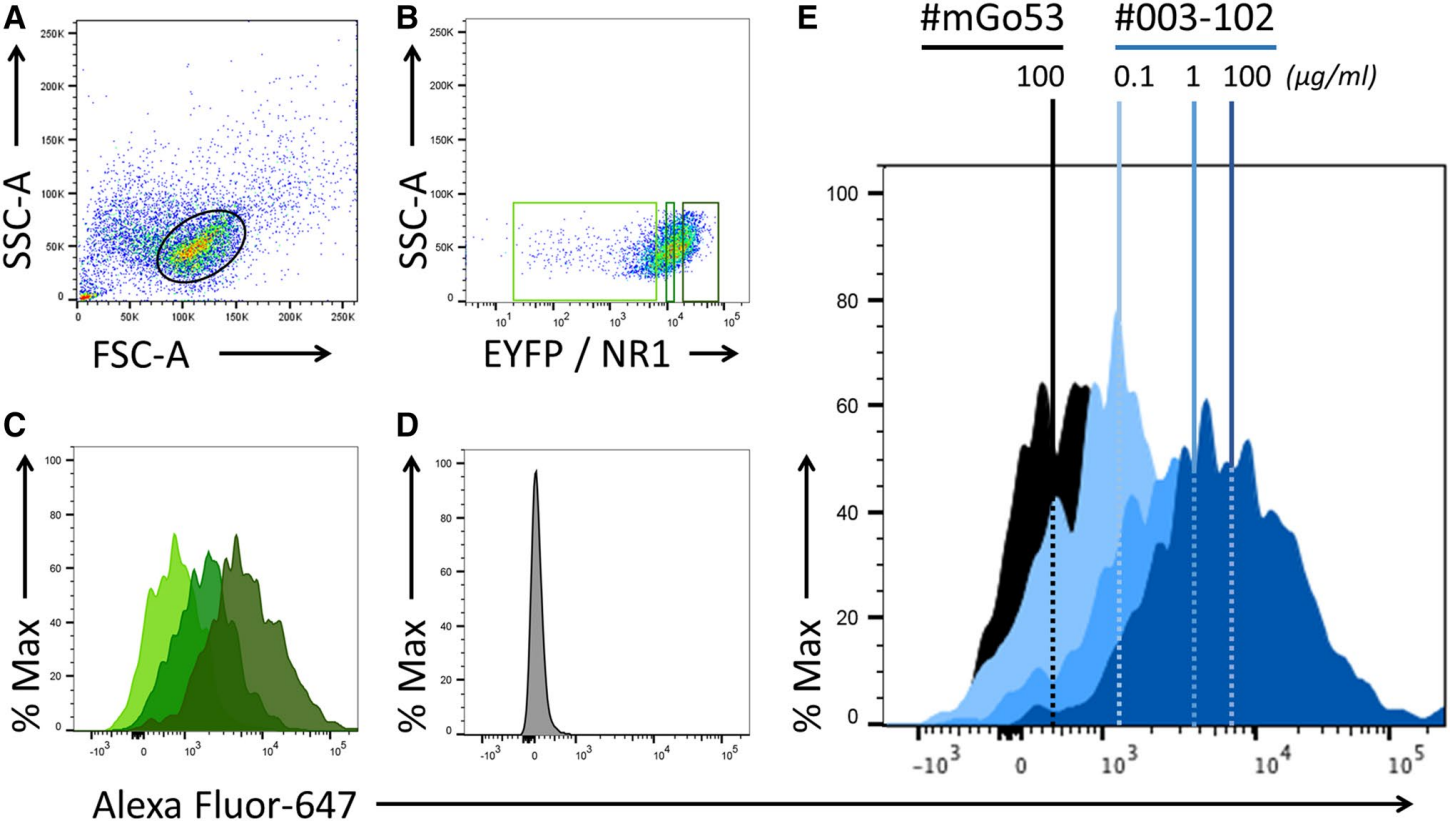
Validation of binding assays using monoclonal human anti-NR1 autoantibodies. NR1–EYFP-transfected HEK293 cells (**a**) were gated for the population with the maximum 20% NR1–EYFP protein expression (dark green rectangle), determined by the fluorescence of EYFP (**b**). EYFP fluorescence correlated well with NR1 expression as determined by staining with the NR1-reactive antibody #003-102 [**c**, lowest/median/highest 20% EYFP-fluorescent populations (gated in **b**) shown in light/medium/dark green, respectively]. In contrast, NR1-reactive antibodies (exemplarily shown for #003-102 at 100 µg/ml) did not bind to control HEK cells transfected with EYFP only (**d**). The NR1-reactive antibody #003-102 showed a concentration-dependent right shift of the fluorescence curves (blue; 0.1, 1 and 100 µg/ml) compared to the background fluorescence (black; 100 µg/ml) of a human control monoclonal antibody #mGo53 [**e**, median fluorescence intensity (MFI) is shown by vertical dotted lines]

For flow cytometry analysis, EYFP expression was used to determine NR1–EYFP protein expression (Fig. 1). To reduce background and increase the signal-to-noise ratio, we restricted our analysis to the relatively homogeneous 20% subpopulation of cells with the highest NR1–EYFP expression. Of these cells, the Alexa Fluor-647 median fluorescence intensity (MFI) was determined, representing the binding of monoclonal NR1 antibodies. Background fluorescence was subtracted from each MFI (Alexa Fluor-647 signal of #mGo53 control antibody). MFI was normalized to reduce inter-experiment variabilities. For this, in every experiment the MFIs of the monoclonal antibodies #003-102 and #007-168 were determined at a concentration of 300 µg/ml, and the mean of both values considered as control value (MFI_control_). This value served as an internal control for all other measurements, similar to a calibration/standard sample in most common assays such as ELISAs. MFI of each sample (MFI_sample_) was then normalized to this value by the equation MFI_normalized_ = MFI_sample_/MFI_control_.

Data from all independent experiments were averaged and standard error of the mean (SEM) calculated. The data were fit with Hill equations using Igor Pro 6.37 (Wavemetrics, OR, USA), resulting in two parameters for quantification of relative antibody affinity: the maximum fluorescence intensity MFI_max_ and the half-maximal binding constant *c*_50_ representing the antibody concentration where 50% of MFI_max_ was reached. Both numbers provide a measure of relative affinity, with higher values of MFI_max_ and lower values of *c*_50_ corresponding to higher antibody affinity.

### Human CSF samples

For determination of NR1 antibody avidity (reflecting the divalent binding of IgG antibodies to their target and the presence of several anti-NR1 antibodies) in human specimen, CSF samples of six patients with NMDAR encephalitis were randomly selected. Total IgG concentration of the CSF was measured with ELISA. CSF of each patient was serially diluted (1:1, 1:3, 1:10, 1:30, 1:100) in FACS buffer and 80 µl was added to 96-well plates. Addition of NR1-expressing HEK cells, staining with secondary antibodies and binding determination with FACS was performed as described above. This experiment was independently repeated three times.

### Control CSF spiked with monoclonal NR1 antibodies

Control CSF was acquired via CSF bolus withdrawal during routine diagnostic in a patient with benign cranial hypertension. No NMDAR antibodies were present in the sample. 20 µl of CSF was added to 96-well plates. 20 µl of either the monoclonal antibody #003-102 or #007-168 diluted in CSF were added to the wells to reach final concentrations ranging from 100 to 0.01 µg/ml and incubated for 20 min. Addition of NR1-expressing HEK cells, staining with secondary antibodies and binding determination with FACS was performed as described above. This experiment was independently repeated three times.

## Results

### Affinity of monoclonal human autoantibodies

NR1–EYFP was expressed as a fusion protein in HEK293 cells. NR1–EYFP-expressing HEK293 cells were incubated with serial dilutions of five monoclonal human NR1 autoantibodies and two isotype-matched non-NR1-reactive control antibodies. Antibody binding to HEK293 cells was quantified via flow cytometry (Fig. 1a–e). EYFP fluorescence correlated well with NR1 expression as determined by monoclonal antibody binding (Fig. 1b, c). No binding of any of the antibodies to control EYFP-transfected HEK cells was detected (Fig. 1d). Compared to control antibodies (Fig. 1e, black), monoclonal NR1 antibody binding led to a concentration-dependent shift of the secondary antibody fluorescence curve (Fig. 1e, blue), with an increase of the median fluorescence intensity (MFI).

**Fig. 2:**
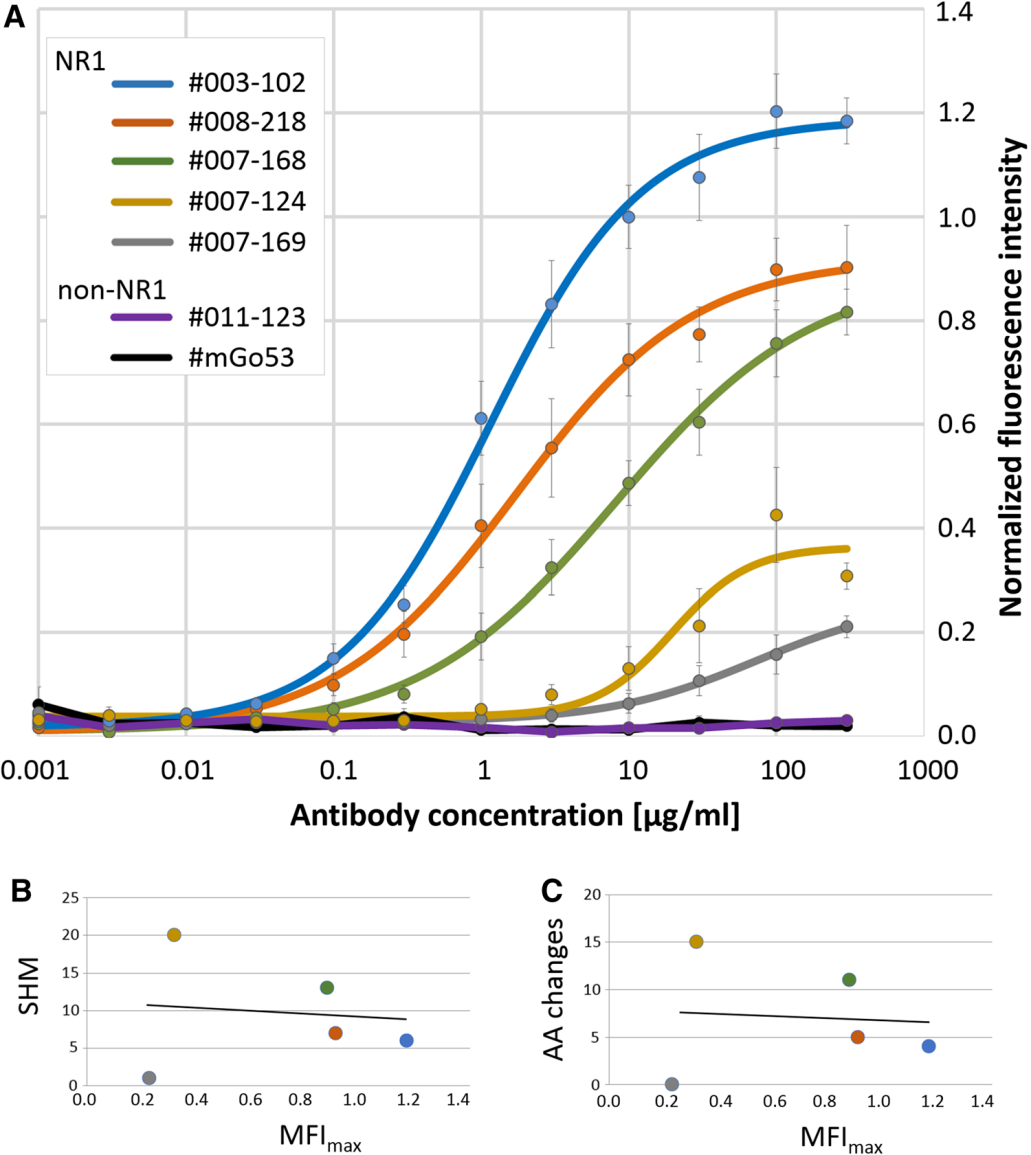
Binding curves of monoclonal human anti-NR1 autoantibodies. MFI of all measurements were normalized and plotted (MFI ± SEM) against the concentration of the monoclonal antibodies, and sigmoid functions with the best fit were generated demonstrating large differences in NMDAR binding (**a**). The MFI_max_ was not correlated with the number of mutations in the antigen-binding site of the NR1 autoantibodies at the DNA (**b**; *SHM* somatic hypermutations) or protein level (**c**; *AA* amino acids)

Normalized MFI of all values were plotted against the antibody concentration (Fig. 2a). Both control antibodies (#011-123, #mGo53) did not show any binding to the NR1–EYFP-expressing cells. The NR1-targeting monoclonal antibodies showed a concentration-dependent sigmoid binding curve, and sigmoid functions with the best fit were calculated (Fig. 2a). Antibody binding curves were different between individual monoclonal NR1 antibodies with saturation plateaus at high antibody concentrations reaching MFI_max_ values of 0.23–1.17. Similarly, the half-maximal concentration *c*_50_ varied between 1 and 74 µg/ml (Table 1). Of the examined clones, #003-102 was the antibody with the highest NR1 affinity, demonstrated by the highest MFI_max_ and the lowest *c*_50_. The MFI_max_ did not correlate with the number of antibody mutations compared to germline configuration at the DNA (Fig. 2b; *R*^*2*^ = 0.017) or protein level (Fig. 2c; *R*^*2*^ = 0.008).

**Table 1:** MFI_max_ and *c*_50_ of human monoclonal antibodies, reflecting their relative affinity to the NR1 protein

Monoclonal antibody	MFI_max_	Binding constant *c*_50_
#003-102	1.17	1.16
#008-218	0.91	1.70
#007-168	0.88	8.17
#007-124	0.32	19.87
#007-169	0.23	74.05

### Binding curves of human CSF samples

**Fig. 3:**
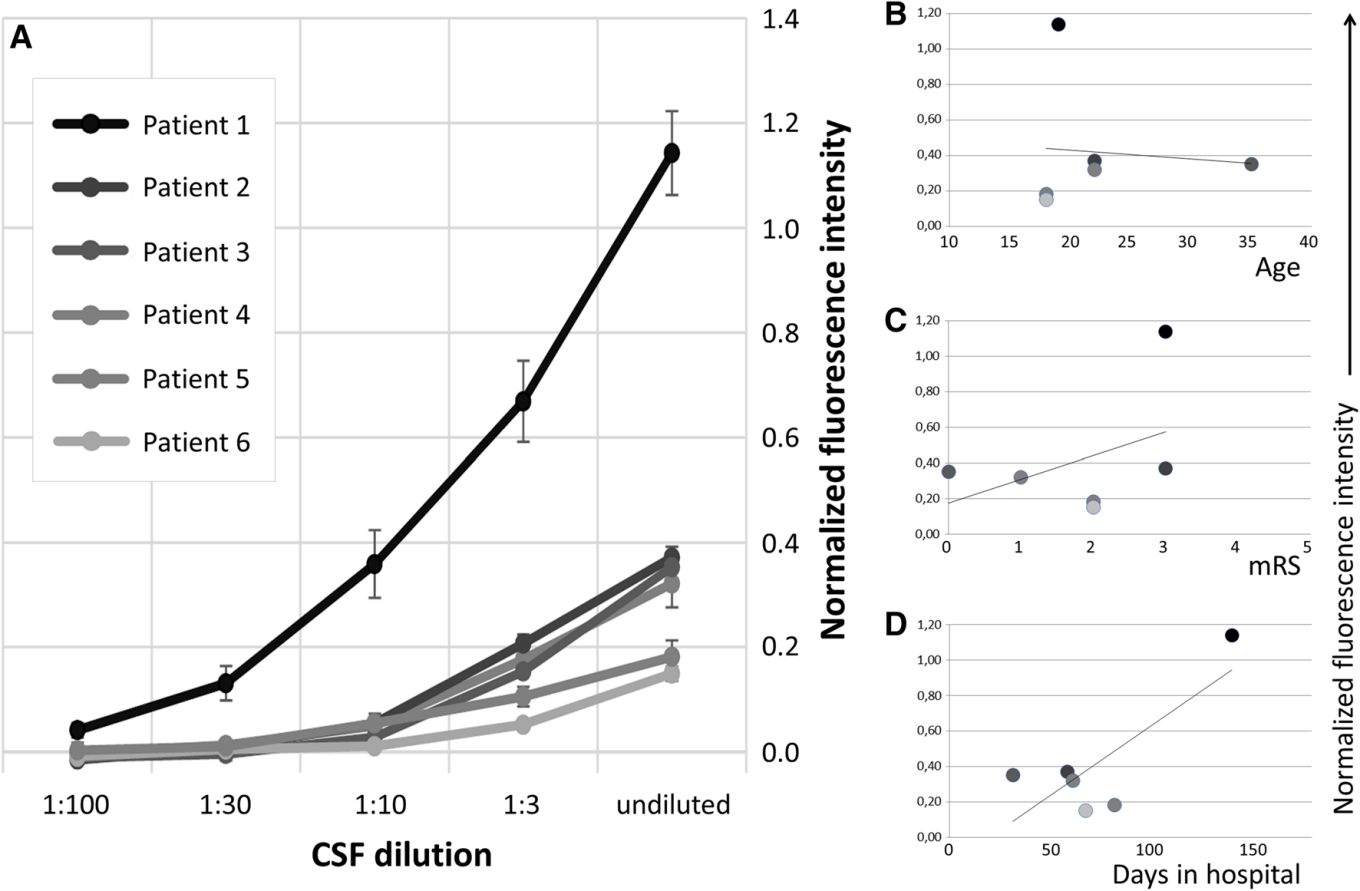
Binding curves of CSF samples from patients with NMDAR encephalitis. Normalized MFI signals (± SEM) show concentration-dependent binding of human CSF samples to NR1 protein (**a**). None of the binding curves reached their MFI_max_ plateau, indicating that CSF NR1 antibody concentrations were clearly below the saturation of the NR1 epitopes and that the binding constant *c*_50_ cannot be calculated in these samples. The MFI did not correlate in this small patient cohort with patient age (**b**), modified Rankin scale at the time of CSF analysis (**c**) and the duration of the hospital stay (**d**)

Human CSF samples contain an undetermined number of low- and high-affinity NMDAR autoantibodies. Using our flow cytometry-based approach, we measured the avidity of this polyclonal mixture to the NR1 protein. For this, CSF from six patients with NMDAR encephalitis was serially diluted and the normalized MFI curves measured (Fig. 3). None of the curves reached their plateau MFI_max_, indicating that the NR1 antibody concentrations in the human samples were far below the saturation of the NR1 epitopes. Therefore, it is not possible to calculate the half-maximal binding constant *c*_50_ in these samples.

The undiluted samples had MFI values between 0.15 and 1.14, representing a normalized NMDAR antibody titer of the respective patient’s CSF with high reproducibility, given the small variations in repeated measurements. Thus, the data indicate that normalization might be an interesting way to allow comparable inter-laboratory quantification of CSF NR1 antibody titers in clinical routine samples of autoimmune encephalitis patients. In this small cohort, no correlations of the MFI were seen with clinical features such as patient age (Fig. 3b; *R*^2^ = 0.008), modified Rankin scale at the time of CSF analysis (Fig. 3c; *R*^2^ = 0.18) and the duration of the hospital stay (Fig. 3d; *R*^2^ = 0.62).

Different to monoclonal NR1 antibodies, it is unclear which concentrations of NR1 antibodies are in the patient’s CSF. To get an estimate of the NR1-specific antibody concentration, we hypothetically assumed that only one monoclonal NR1 antibody is present in the CSF. With this assumption, we calculated how much of each monoclonal NR1 antibody would be required to reach the MFI of the undiluted CSF sample (Table 2). For example in patient 3, the CSF MFI of 0.35 equaled a concentration of 0.39 µg/ml of antibody #003-102. In contrast, 127.6 µg/ml of antibody #007-124 would be required which by far exceeded the total IgG concentration in this CSF sample (Table 2). In some instances, the MFI_max_ plateau of low-affinity monoclonal antibodies precluded the concentration required for the CSF MFI, such as #007-169 for patients 1–4.

**Table 2:**
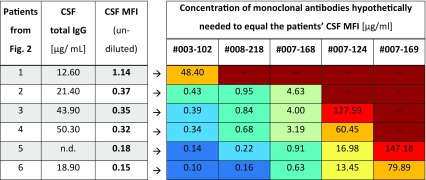
Concentrations of monoclonal human NR1 autoantibodies calculated from binding curves to cause an MFI that is identical to the MFI of undiluted CSF samples

The heat map (right) shows for each monoclonal antibody which concentration would hypothetically be required to reach the fluorescence intensity of undiluted CSF for each patient (left). Low concentrations of high-affinity monoclonal antibodies (dark blue to turquoise) are sufficient to explain the MFI of most undiluted CSF samples from patients with NMDAR encephalitis (e.g., #003-102 for patients 2–6). In contrast, concentrations of low-affinity antibodies (e.g., #007-169, extreme right lane) needed to receive the same signal would often exceed the total IgG concentration in the patients’ CSF (antibody concentrations in orange to dark red) and can therefore not explain the antibody signal in the patient sample

*n.d*. not determined

In one patient (#6), the MFI of undiluted CSF was so high, that our highest-affinity NR1-reactive monoclonal human antibody (#003-102) would be required in a concentration exceeding the total CSF IgG, while none of the other antibodies can even reach such MFI (Table 2). Thus, this patient’s CSF must contain NR1-targeting antibodies of yet higher affinity. We therefore conclude that the CSF signal is predominantly represented by high-affinity antibodies, according to our calculations likely in a concentration range of NR1 antibodies between 0.1 and 5 µg/ml, roughly reflecting 1–10% of the total IgG in CSF (Table 2). These calculations teach, on the contrary, that even high amounts of low-affinity NR1 antibodies can easily remain undetected in state-of-the-art diagnostics such as cell-based assays.

### Effect of CSF composition on NR1 autoantibody binding curves

**Fig. 4:**
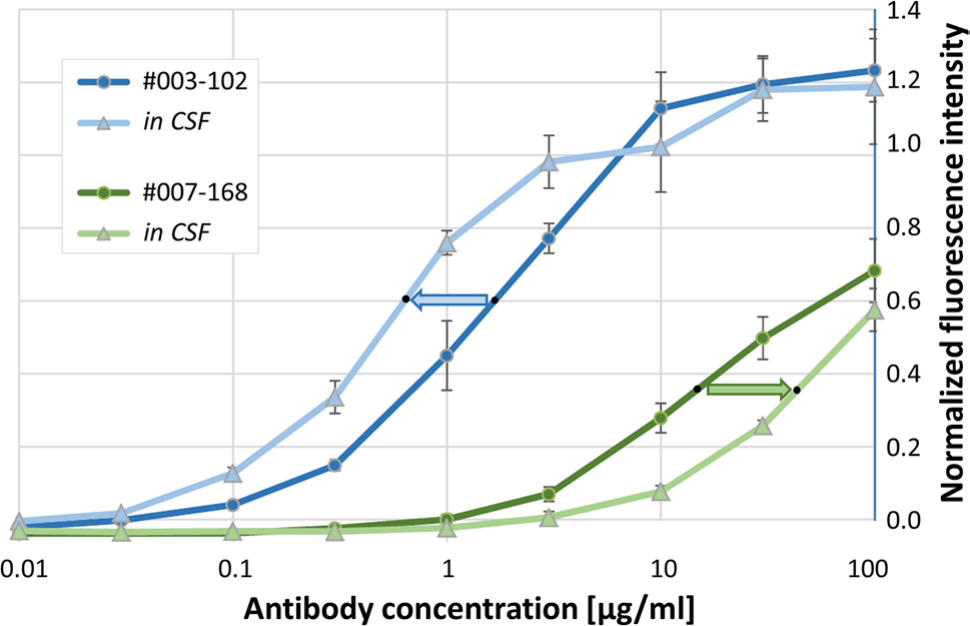
Binding curves of human monoclonal NR1 autoantibodies diluted in CSF. The binding constant *c*_50_ was markedly reduced (blue arrow) or increased (green arrow) depending on which monoclonal NR1 antibody was diluted in the identical control CSF

In addition to affinity, further intrinsic biophysical properties of the NR1 antibodies might contribute to their target binding and pathophysiological functions. We therefore examined whether serial dilutions of monoclonal human NR1 autoantibodies in physiological CSF resulted in changes to the affinity curves. Indeed, after diluting the high-affinity NR1 antibody #003-102 in the CSF of a patient with benign intracranial hypertension after exclusion of NMDAR autoantibodies, there was a marked left shift of the curve with approximately threefold reduction of *c*_50_, i.e., the antibody concentration required to give the same MFI signal was decreased (Fig. 4, blue arrow). In contrast, the identical CSF spiked with the monoclonal human NR1 antibody #007-168 led to a marked right shift of the binding curve with an approximately threefold increase of *c*_50_ (Fig. 4, green arrow). Thus, depending on the type of NR1 antibody, components of the human CSF, e.g., albumin and globulins, might affect the fluorescence signal in established assay systems in opposite ways.

## Discussion

The present study demonstrated that patient-derived monoclonal NMDAR autoantibodies have variable binding curves that can be accurately measured with flow cytometry utilizing NR1-expressing HEK293 cells. Binding curves allow calculation of the maximum binding capacities (corresponding to MFI_max_ at saturation plateaus) and half-maximal antibody concentrations *c*_50_. Both values reflect the affinity of monoclonal antibodies, indicating that human antibodies can be ordered by their relative affinity, even though exact quantification of the affinity of antibody–antigen interaction (usually expressed by the dissociation constant *K*_d_) would require further data such as the free antibody concentration not addressed in this study [6].

The data add another view on the interpretation of antibody titers in clinical samples which only partially correlate with clinical disease [1]. It is known from related neurological autoimmune disorders that antibody affinity might determine disease severity. For example, in Guillain–Barré syndrome, the affinity of anti-GM1 antibodies was associated with disease onset, suggesting an affinity threshold for disease induction [3]. Similarly, the affinity of NMDAR antibodies should be further analyzed in large patient cohorts to determine whether the combination of antibody titer and affinity provides better correlation with disease and prognosis. This might then be extended to even more sophisticated technical methods such as surface plasmon resonance, which would allow real-time affinity determination without the need for antibody labeling.

Along these lines, even titers of NR1 antibodies are hardly comparable between laboratories, likely related to variable protocols for the diagnostic assay. Variables include the expression levels of NR1 protein in cell-based assays or neuronal cells, incubation times of the cells with human samples, secondary detection antibodies and experience of the rater for often subjective determination of fluorescence intensity, among others. In clinical routine, it is therefore often difficult to judge the effect of immunotherapy based on proxy antibody titers, even if samples were analyzed in the same laboratory. Our study showed that normalization of the data might be an elegant way to overcome this problem. Comparing the binding of a given human CSF sample to an assay standard comprised of exactly determined concentrations of monoclonal human antibodies, the NR1 antibody titer was reproduced with minimal variation in repeated measurements. High reproducibility and easy handling of standard antibody solutions suggests that normalization will allow comparable inter-laboratory quantification of CSF NR1 antibody titers in clinical routine samples of autoimmune encephalitis patients. Similar normalizations were devised for other clinical routine assays, with the INR (International normalized ratio) for evaluation of the patient’s coagulation being one of the most prominent examples, e.g., in patients on warfarin. Normalizing the prothrombin time to control plasma made it possible to compare coagulation between laboratories worldwide.

Although the patient CSF contains several monoclonal NMDAR antibodies, the antibody signal seen in routine diagnostic assays is likely related to the high-affinity antibodies. Low-affinity antibodies would be required in such high concentrations for reaching the binding intensity of CSF samples that their IgG concentration exceeded the total IgG in the sample. Therefore, low-affinity NR1 antibodies can easily go undetected in cell-based assays, even though it is unclear yet whether only such low-affinity (without additional high-affinity) antibodies can be present in patients. If so, then the intriguing question arises of whether low-affinity antibodies may still account for clinical symptoms, in particular if produced by plasma cells in the brain parenchyma where the local antibody concentrations might be high. Prolonged exposure might still produce synaptic changes and thus result in subtle clinical abnormalities which were until now not considered as NR1 autoimmunity.

We further found that the dilution of monoclonal human NR1 autoantibodies in control CSF can affect the binding curves in opposite ways. It is not clear from this study whether certain CSF proteins, e.g., albumin or globulins, the most abundant proteins in non-inflammatory CSF, bind to the NR1 antibodies differently and interfere with their receptor interaction. Besides affinity, potential determinants include the antibody polyreactivity, not assessed in this study. We do not assume that the protein binding is mediated via Fc receptors as the examined monoclonal antibodies had the identical Fc part. The data suggest that changes in the protein concentration and composition (e.g., with encephalitis-related increase in the CSF or with therapeutic apheresis-related decrease in blood and CSF) can further influence the NR1 antibody function in NMDAR encephalitis.

Taken together, we identify reproducible and measurable biophysical factors of NMDAR autoantibodies that should be further evaluated in large patient cohorts. Data can immediately improve clinical routine assays for antibody detection, might provide surrogate markers for monitoring of disease course and prognosis, and should be expanded to other forms of autoimmune encephalitides.
